# Chronotypes and their Association with Obesity-Related Lifestyle Behaviors among Young Female Adults

**DOI:** 10.3390/ijerph20021305

**Published:** 2023-01-11

**Authors:** Rawan A. Al Abdullatif, Shaea Alkahtani, Graham Finlayson, Maha H. Alhussain

**Affiliations:** 1 Department of Food Science and Nutrition, College of Food and Agriculture Sciences, King Saud University, Riyadh 11451, Saudi Arabia; 2 Department of Exercise Physiology, College of Sport Sciences and Physical Activity, King Saud University, Riyadh 11451, Saudi Arabia; 3 School of Psychology, Faculty of Medicine & Health, University of Leeds, Leeds LS2 9JT, UK

**Keywords:** chronotype, females, dietary intake, sleep, physical activity

## Abstract

Circadian rhythms have emerged as key influences on lifestyle behaviors. Circadian rhythms vary inter-individually, and people can be stratified by circadian preference, known as their chronotype, from extreme morning types to extreme evening types. Young adults undergo chronotype changes that involve shifts from morning to evening types. We aimed to examine the association between chronotype and obesity-related lifestyle behaviors, including dietary intake, physical activity, and sleep patterns, among young females. A total of 387 college female students aged 18–25 years completed this cross-sectional study. The participants were classified into three groups (morning, intermediate, and evening types) according to the Morningness–Eveningness Questionnaire (MEQ; long version) score. Each individual’s anthropometry and body composition were measured. Dietary intakes were assessed using a 24-h dietary recall. The Bouchard Three-Day Physical Activity Record was used to assess physical activity levels. Sleep patterns were evaluated using the Pittsburgh Sleep Quality Index (PSQI) and Epworth Sleepiness Scale (ESS). The differences between chronotype groups were tested using a Chi-square test and one-way ANOVA. The chronotypes were significantly associated with sleep quality (*p* = 0.010) and daytime sleepiness (*p* = 0.035). However, no significant associations between dietary intake, physical activity, and sleep duration with the chronotypes were found. Our results show that both sleep quality and daytime sleepiness were associated with the chronotypes. Further research is warranted to identify the potential bidirectional associations between circadian rhythms and lifestyle behaviors among different age groups.

## 1. Introduction

Obesity is a complex condition that adversely affects quality of life. Extreme adiposity causes negative metabolic responses resulting in comorbidities and early mortality [1,2]. With the sharp increase in the prevalence of obesity in the last few decades, modifiable lifestyle behaviors have received growing attention. Circadian rhythms have emerged as key contributors to obesity [3,4]. Circadian rhythms refer to the synchronicity between endogenous biological rhythms such as hormone secretion, exogenous rhythms such as the light–dark cycles of the twenty-four-hour day, and behavioral rhythms such as a person’s sleep/wake sequence. They affect many biological functions, including endocrine, metabolic, neuronal, and behavioral functions [5]. These rhythms’ alignment allows the body to function efficiently because hormones linked to habitual functions prepare the human body for its expected behaviors [6]. The misalignment of these rhythms has been associated with an increased risk of adverse health conditions, including obesity [6,7] and metabolic syndrome [8]. Moreover, it has also been linked to cardiometabolic illness [9].

Circadian rhythms vary among individuals, and people may be classified based on their circadian preferences, known as chronotypes, into morning-type (M-type), evening-type (E-type), and intermediate-type (I-type) individuals [10]. Numerous studies have shown differences between chronotypes with regard to their physiological outcomes [11,12].

Understanding the effect of a chronotype on lifestyle behaviors such as dietary intake, physical activity (PA), and sleep patterns could inform preventative health efforts. The evidence suggests that the E type is correlated with a higher body mass index (BMI), unhealthy dietary intake, and physical inactivity [13,14,15]. Moreover, a growing body of evidence suggests that one of the main changes in modern societies that leads to the pathogenesis of obesity involves a pervasive rise in voluntary sleep curtailment [16]. These disorders of sleep are correlated with decreased glucose and lipid metabolism, increased hunger, and broad changes in the hormonal signals that regulate food intake [17]. Therefore, suggesting potential pathways through which the chronotype can affect obesity is important.

There are limited studies quantifying the extent of the association between chronotypes and lifestyle behaviors. Moreover, no studies have reported such associations among Saudi females. Therefore, this study aimed to investigate the associations between chronotypes and obesity-related lifestyle behaviors, including dietary intake, PA, and sleep patterns, among Saudi young female adults.

## 2. Materials and Methods

### 2.1. Study Design and Participants

This cross-sectional study was conducted among Saudi female students (aged 18–25 years) at King Saud University, Riyadh, Saudi Arabia, between October 2020 and June 2021, using a convenience sampling method. The study advertisement was distributed both in paper and electronically to the students. Healthy students without any physical or mental illness and not taking any medications other than oral contraceptives were eligible to participate in the study. The exclusion criteria were as follows: pregnant and lactating females, professional athletes, and those who were out of the age range. The total sample size consisted of 387 female students.

We calculated the sample size as being within 0.05 of the population proportion with a 95% confidence level. We assumed the population proportion to be 0.50, because this proportion gave the maximum possible required sample size [18]. The study received ethical approval from the Institutional Review Board at King Saud University (No. E-20-4656). The participants provided informed consent prior to the study.

### 2.2. Assessments

#### 2.2.1. Chronotype

The ME Morningness–Eveningness Questionnaire (MEQ) is the most widely used tool for identifying chronotypes [10] (See Supplementary Materials). We used the long version of the MEQ, which is composed of nineteen items related to sleeping and waking hours, schedule preferences, and subjective alertness at different hours of the day. Under these items, the participants were required to choose one of four answers (e.g., “when you have no commitments the next day, at what time do you go to bed compared to your usual bedtime?”). The answers were: (4) seldom or never later; (3) less than 1 h later; (2) 1–2 h later; (1) more than 2 h later. The total scores ranged from 16 to 86 points. According to the scores, the chronotypes were divided into three types, as follows: E-type (16–41 points); I-type (42–58 points); M-type (59–86 points).

#### 2.2.2. Anthropometric Measurements

Height was measured to the nearest 0.5 centimeters (cm) using a wall-mounted stadiometer. Weight was measured via a bioelectrical impedance analysis (BIA) (InBody 770, Inbody Co., Ltd., Seoul, Republic of Korea) with an empty bladder, light clothes, and no shoes. The BMI was calculated as the weight divided by the square of the height (kg/m^2^). The waist and hip circumferences were taken using a non-stretch measuring tape to the nearest 0.5 cm and the waist–hip ratio (WHR) was calculated [19]. The body composition was assessed using a BIA. The visceral fat (cm^2^), percentage of body fat (BF%), and skeletal muscle mass (SMM) were also assessed. The appendicular lean mass (ALM) was determined by calculating the sum of the lean tissue in the arms and legs divided by the height squared (ALM/h^2^) or BMI (ALM/BMI). The skeletal muscle index (SMI) was calculated by dividing the SMM (kg) by the weight. All measurements were taken by the same investigator.

#### 2.2.3. Dietary Intake

Face-to-face interviews were conducted to obtain 24-h dietary recall information for a typical day. Food measuring cups and spoons were used to help participants estimate the portion sizes of the foods and beverages consumed. The dietary intake data were analyzed using the ESHA Food Processor (ESHA Research Inc., Salem, OR, USA). Daily energy and macronutrient intakes were assessed.

#### 2.2.4. Physical Activity

PA was determined with the use of the Bouchard Three-Day PA Record, which was designed to estimate participants’ energy expenditure [20]. Participants used this to self-record daily activities over three days (two weekdays and one weekend day). In the activity record, a day was divided into ninety-six periods of fifteen minutes each. For each fifteen-minute period, the energy expenditure was qualified on a scale from 1 to 9.

#### 2.2.5. Sleep Pattern

The habitual sleep duration was estimated subjectively based on sleep diary records for three days (two weekdays and one weekend day). The participants were asked to fill out the diary every morning when they woke up, every night when they went to bed, and every time they napped. They were instructed to keep the diary beside their beds to make sure they did not forget to fill it in. We obtained the total sleep duration by adding up all sleep hours and dividing the number by three to obtain the average.

Sleep quality was assessed using the Pittsburgh Sleep Quality Index (PSQI), a validated nineteen-item questionnaire with questions about sleep over the past month, including subjective sleep quality, sleep latency, sleep duration, habitual sleep efficiency, sleep disturbances, use of sleep medication, and daytime dysfunction. The seven component scores ranging from 0 to 3 are summed to yield the total PSQI score, which ranges from zero to 21 points. A total PSQI score > 5 is associated with poor sleep quality [21].

Daytime sleepiness was assessed using the Epworth Sleepiness Scale (ESS). The ESS was introduced by Johns [22] and is composed of an eight-item questionnaire that assesses the severity of daytime sleepiness in various situations. Score ranging from zero to 9 are defined as normal, while 10 to 24 indicate daytime sleepiness.

#### 2.2.6. Demographic Data

An administered self-reported questionnaire was used to collect sociodemographic information, including age, marital status, monthly household income, college, academic year, and evening job status.

### 2.3. Statistical Analysis

All statistical tests were performed using IBM SPSS Statistics version 28 (IBM Corp., Armonk, NY, USA), and MS Excel was used for data management. The data were checked for normality using the Kolmogorov–Smirnov test. The descriptive statistics are presented as means ± SD for continuous and normally distributed data, medians (Q1–Q3) for non-normally distributed data and frequencies, and percentages for categorical data. The differences between chronotype groups were examined using a one-way ANOVA, followed by Scheffe’s post hoc test, as well as the Kruskal–Wallis test for continuous variables. Chi-squared and Fisher–Freeman–Halton exact (for small frequencies) tests were used for categorical variables. A linear regression analysis was used to analyze the association between the chronotype and BMI. Statistical significance was set at *p* < 0.05 for all statistical tests.

## 3. Results

### 3.1. Participant Characteristics

The participant demographic characteristics are shown in Table 1. The participants were on average 20.70 ± 1.62 years of age, and the majority of the sample was single (96.6%), with a monthly income of more than 20,000 Saudi Riyal (31.1%). Students from health colleges made up 40% of the sample. Most of the participants (98.1%) did not have an evening job after university.

### 3.2. Chronotype

**Figure 1:**
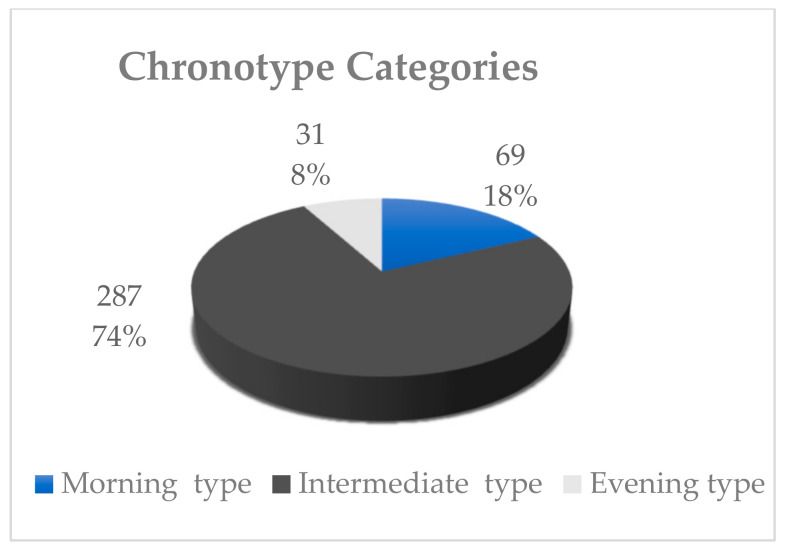
Chronotype categories of the study participants (n = 387).

Figure 1 shows the chronotype categories of participants, of which 74.2% were I-type, 17.8% were M-type, and 8% were E-type.

#### 3.2.1. Chronotype and Anthropometric Measurements

**Figure 2:**
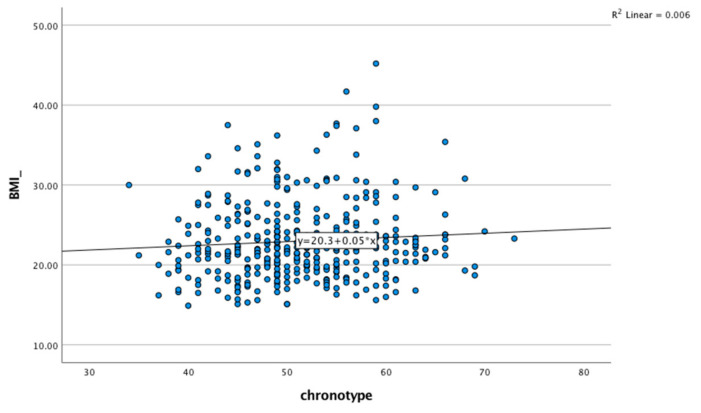
Linear regression analyses between chronotypes and BMI scores.

The anthropometric measurements for the study participants according to chronotype are shown in Table 2. The average BMI for the three chronotype categories was within the normal range. There were no significant differences between the chronotype categories with regard to the BMI or the other anthropometry characteristics. The linear regression analyses between chronotypes and BMI scores showed that y = 20.3 + 0.05x, R^2^ = 0.006. (Figure 2).

#### 3.2.2. Chronotype, Energy Intake, and Energy Expenditure

Table 3 displays the energy intake scores according to chronotype. The average energy intake for all participants was 1335.1 ± 409.9 Kcal/d. There were no significant differences by chronotype in terms of the total energy and macronutrient intakes.

The mean energy expenditure for all participants was 2391.7 ± 659.1 Kcal/d (Table 3). No significant differences were observed among chronotype categories related to energy expenditure.

#### 3.2.3. Chronotype and Sleep Pattern

The sleep patterns for all study participants and according to chronotype categories are shown in Table 4. The majority of the participants had poor sleep quality and normal sleepiness. The sleep diaries indicated that the mean sleep duration for all participants was 8.30 ± 1.82 h. Regarding the chronotype groups, there were significant associations between the chronotypes and sleep quality (PSQI) (Chi-squared, *p* = 0.007). Furthermore, there were significant differences in sleep quality scores between the chronotype groups (one-way ANOVA, *p* = 0.003), and the M-type group had better sleep quality with a lower score as compared with the I-type and E-type. In terms of daytime sleepiness, there was a significant association between daytime sleepiness and each chronotype (Chi-squared, *p* = 0.035). The sleepiness scores also showed significant differences between chronotype groups (one-way ANOVA, *p* = 0.034). The M-type group had a lower score than the I-type group. On the other hand, no significant differences between chronotype categories in terms of sleep duration were noted.

## 4. Discussion

This study aimed to examine the associations between chronotypes and dietary intake, physical activity, and sleep patterns among young females. Overall, the results of this study showed that the chronotype was significantly associated with sleep quality and daytime sleepiness. However, there was no significant association of the chronotype with dietary intake or energy expenditure.

In the present study, the vast majority of the participants had the I type. In line with these findings, a local study [23] conducted among young adult Saudis reported that most of the study sample had the I type. Similar findings were also reported in previous studies conducted in Western populations [24,25].

Evidence suggests that the E type is independently associated with obesity development [26,27]. Sun et al. [15] showed that a trend for the E type was associated with obesity. Culnan et al. [28] found that E-type individuals showed significantly greater BMI gains compared with M-type and I-type individuals. In the current study, no significant differences in anthropometric variables were found between the chronotype categories. A previous study examined the associations between chronotypes and the anthropometric variables in seventy-two medical residents and reported that the chronotype was not associated with anthropometry [29]. Studies examining the associations between chronotypes and BMI scores in different regions and with different ages, genders, and sample sizes have reveled inconsistent results. Some studies have shown that age and gender have a role in influencing the chronotype [24,30]. Further studies with larger sample sizes with different ages and genders are needed to confirm the association between the chronotype and obesity.

The BMI scores in this study were in the normal range, despite showing a lower average daily energy intake compared with the energy requirements. The under-reporting of the daily energy intake is a common and acknowledged source of measurement error in the assessment of food intake [31]. The data collected using a twenty-four-hour dietary recall, a memory-based dietary assessment method, might depend on the respondent’s honesty, memory, and ability to estimate their past dietary intake.

Sleep patterns can be influenced by factors such as an individual’s age, work schedule, and circadian rhythm [32]. The results of the current study showed a significant association between the chronotype and sleep quality. The participants who were classified as M-type had better sleep quality compared with I-type and E-type groups. These findings align with previously reported results, wherein relationships between chronotypes and sleep quality were reported [33,34]. An individual’s type of work and a student’s major can affect the individual chronotype and sleep pattern. Rique et al. [35] examined the chronotypes and their association with sleep quality among 221 medical students and found an association between the chronotype and sleep quality. In contrast, Culnan et al. [28] found that the chronotype was not associated with sleep quality among 137 college freshmen. A possible explanation for the differences between our findings and Culnan et al.’s [28] findings is that the latter study included freshmen, who were younger than our participants.

Regarding the association between the chronotype and daytime sleepiness, we found that all three groups had normal daytime sleepiness levels, although the M-type group had a better score. The research done by Rique et al. [35] found that medical students’ chronotypes were not associated with increased daytime sleepiness, in contrast with the results of our study. Similarly, the research done by Upadhyay et al. [36] on the association between daytime sleepiness and the chronotype in undergraduate medical and paramedical students found a significant association between daytime sleepiness and the chronotype. It is possible that students with poor sleep quality experience daytime sleepiness.

Recently, there has been a shift in interest in the consequences of sleep duration decreases for other organs and physiological systems. The evidence has shown that sleep may affect an individual’s energy balance and cause obesity [37,38]. In the current study, we found no significant association between the chronotype and sleep duration, and the mean sleep duration was in the normal range. Our results are in agreement with Martin et al. [39], who studied student workers aged nineteen to twenty-one. Social zeitgebers may have an effect on the sleep-linked problems of the student population.

The current study found no significant association between the chronotype and PA. Consistent with our observations, other studies have reported no significant associations between the chronotype and PA [28,34].

In general, the chronotype may affect an individual’s food choices and eating habits [29,40]. The studies about the association between the chronotype and dietary intake were inconsistent. Many studies did not find significant differences in total energy intake levels between chronotypes [40,41,42], and the current study found the same findings. Moreover, in the present study, there was no significant association between the chronotype and carbohydrate intake [43], but negative associations were found with the others [34,44]. Some studies indicated that the protein intakes of E-types were found to be lower in terms of macronutrients [44,45]. However, the present study did not find a significant association between protein intake and chronotype. Additionally, previous studies indicated that E-types consume more fat [34,44]. On the contrary, our study found no significant association between fat intake and chronotype. Food intake is determined by multiple factors, whether physiological or environmental, such as the access and availability of food [46], and by emotional factors such as anxiety and stress levels; studies analyzing all of these factors together in individuals of different chronotypes allow us to understand the influence of the chronotype on the choice of food intake.

Several limitations of the current study have to be considered when interpreting the results. The cross-sectional design did not allow us to draw any directionality in the relationships. In addition, we used a twenty-four-hour dietary recall which is sufficient to detect differences between M-type and E-type individuals but may be inadequate to show the usual dietary intake of the individuals. Another limitation of the study may be the nature of the self-reported data, including for the MEQ, PA, PSQI, ESS, and sleep duration data. Possible memory and reporting biases may have affected the data to some extent; however, this is extremely difficult to assess. Furthermore, the phenomenon that health-conscious people are more likely to participate in health surveys may have affected our results. Those who were overweight may have chosen not to participate due to the sensitivity and stigma that accompany obesity. This may have been especially relevant for young females, as most of the participants were of normal weight. Further studies are needed among females in different age groups (adolescents, middle-aged and older adults) with and without metabolic syndrome.

## 5. Conclusions

In conclusion, the present study showed that both sleep quality and daytime sleepiness were associated with the chronotype. On the other hand, there was no association between energy intake and energy expenditure with the chronotype. In view of these findings, it is important to consider the relationships between chronotypes and sleep in clinical and research sittings. Future research should use objective measures of sleep and PA (i.e., actigraphy) in combination with subjective measures in order to obtain a clearer picture of how these behaviors relate to the chronotype. Furthermore, accurate estimations of the dietary intake are required. Further research with a larger sample size is warranted to identify the potential bidirectional associations between circadian rhythms and lifestyle behaviors among different age groups.

## Figures and Tables

**Table 1:** Characteristics of the study participants (n = 387).

Variables	Mean ± SD/N (%)
Age (Year)	20.70 ± 1.62
Social status Single Married	374 (96.6) 13 (3.3)
Monthly income (SR) <5000 5000–10,000 10,100–20,000 >20,000	50 (12.9) 106 (27.4) 111 (28.6) 120 (31.1)
College Health colleges Science colleges Humanities colleges	157(40) 131(34) 99 (26)
Academic year 1 2 3 4 5	39 (10.1) 157 (40.5) 81 (20.9) 105 (27.2) 5 (1.3)
Do you have an evening job? No Yes	380 (98.2) 7 (1.8)

Data presented as means ± SD and N (%). SR: Saudi Riyal equivalent to $0.27.

**Table 2:** Anthropometric measurements for the study participants (n = 387).

	Chronotype				
Characteristics	All	M-Type	I-Type	E-Type	*p* Value ^1^
	(n = 387)	(n = 69)	(n = 287)	(n = 31)	
Weight (kg)	57.6 ± 12.8	55.3 (50.5–61.8)	55.1 (49.1–64.3)	53.3 (47.4–60.9)	0.535
High (cm)	157.9 ± 7.8	158 (154.2–161)	158 (154.4–161.5)	159.7 (155.5–163.5)	0.411
BMI (kg/m^2^)	22.9 ± 4.9	22.3 (20.4–23.8)	22.1 (19.7–25.5)	21.2 (18.65–23.4)	0.188
WC (cm)	63.4 ± 9.1	67 (63–71.5)	66.4 (63–72)	64 (59.9–69.5)	0.191
HC (cm)	97.7 ± 10.8	97 (91–102)	96 (91–104)	95 (88.25–99.0)	0.433
Waist-hip ratio	0.7 ± 0.1	0.7 (0.7–0.7)	0.7 (0.67–72)	0.7 (0.66–0.72)	0.420
Visceral fat (cm^2^)	108.9 ± 50.1	99.7 (72.6–140.6)	100.9 (71.6–138.7)	88.4(59.9–125.4)	0.347
Body fat (%)	36.6 ± 7.7	36.7 ± 7.02	36.7 ± 7.5	34.5 ± 7.6	0.312
SMM (kg)	19.1 ± 2.9	18.9 (17–20.2)	18.5 (17–20.8)	18.7 (17.2–20)	0.899
ALM/h^2^ (kg/m^2^)	5.7 ± 1.9	5.5 (5.2–5.8)	5.5 (5.1–6.1)	5.3 (4.9–6.1)	0.494
ALM/BMI	0.6 ± 0.1	0.6± 0.1	0.6 ± 0.09	0.6 ± 0.1	0.174
SMI (kg/m^2^)	33.6 ± 3.9	33.6 ± 3.9	33.5 ± 3.9	34.6 ± 3.9	0.357

Data are presented as medians (Q1–Q3) and means ± SD; ^1^ *p* values significant < 0.05; *p* values tested using a one-way ANOVA (for normally distributed data) and Kruskal–Wallis test (for non-normally distributed data). Abbreviations: BMI, body mass index; WC, waist circumference; HC, hip circumference; SMM, skeletal muscle mass; ALM, appendicular lean mass; SMI, skeletal muscle index.

**Table 3:** Energy intake and energy expenditure rates for the study participants (n = 387).

	Chronotype				
Characteristics	All	M-Type	I-Type	E-Type	*p* Value ^1^
	(n = 387)	(n = 69)	(n = 287)	(n = 31)	
Total energy intake (Kcal/d)	1335.1 ± 409.9	1348.1 ± 366.5	1337.2± 422.5	1285.9 ± 390.2	0.771
Carbohydrate (g/d)	157.8 ± 56.9	161.4 ± 61.9	158.4 ± 57.2	143.7 ± 40.6	0.328
Carbohydrate (energy%)	47.5 ± 10.5	47.6 ± 11.9	47.6 ± 10.1	46.2 ± 10.9	0.775
Protein (g/d)	52.3 ± 24.3	50.9 (39.7–60.9)	47.8 (35.7–66.0)	43.4 (33.0–61.3)	0.661
Protein (energy%)	15.7 ± 6.2	14.9 (12.1–16.9)	14.4 (11.8–18.2)	14.3 (11.8–19.5)	0.984
Fat (g/d)	57.3 ± 24.2	54.3 (43.1–71.1)	54.3 (38.7–70.2)	50.1 (39.1–73.9)	0.893
Fat (energy%)	38.3 ± 9.9	38.6 ± 10.9	38.1 ± 9.6	39.5 ± 10.9	0.747
Energy expenditure (Kcal/d)	2391.7 ± 659.1	2442.6 ± 668.1	2405.5 ± 669.8	2151.3 ± 482.2	0.097

Data are presented as medians (Q1–Q3) and means ± SD; ^1^ *p* values significant < 0.05; *p* values tested using a one-way ANOVA (for normally distributed data) and Kruskal–Wallis test (for non-normally distributed data).

**Table 4:** Sleep patterns of the study participants (n = 387).

	Chronotype				
Characteristics	All	M-Type	I-Type	E-Type	*p* Value ^1^
	(n = 387)	(n = 69)	(n = 287)	(n = 31)	
Sleep quality (PSQI)					
good sleep quality	70 (18.1)	21 (30.0)	47 (67.1)	2 (2.9)	0.007
poor sleep quality	317 (81.9)	48 (15.14)	240 (75.7)	29 (9.1)	
Sleep quality (PSQI) score	6.9 ± 2.7	6.0 ± 2.7 (ab) *	7.1 ± 2.7 (a)	7.8 ± 2.7 (b)	0.003
Sleepiness (ESS)					
normal daytime sleepiness	283 (73.1)	57 (20.1)	200 (70.7)	26 (9.2)	0.035
excessive daytime sleepiness	104 (26.9)	12 (11.5)	87 (83.7)	5 (4.8)	
Sleepiness score	6.8 ± 4.1	5.6 ± 3.9 (a)	7.1 ±4.1 (a)	6.8 ± 3.6	0.034
Sleep duration (h)	8.3 ± 1.8	8.1 ± 2.0	8.4 ± 1.8	7.9 ± 1.6	0.212

Data presented as N (%), means ± SD; ^1^ *p* values significant < 0.05. * The same letter for two groups means that there was a significant difference using Scheffe’s post hoc test. Categorical data tested by Chi-square or Fisher–Freeman–Halton exact test (for small frequencies). Continuous data tested using a one-way ANOVA followed by Scheffe’s post hoc test. Abbreviations: PSQI, Pittsburgh Sleep Quality Index; ESS, Epworth Sleepiness Scale.

## Data Availability

These and other supplementary data that are related to this study’s materials are available in our archives of SPSS and the respective syntaxes. Additionally, this work was not preregistered (not applicable).

## Supplementary Materials

The following supporting information can be downloaded at: https://www.mdpi.com/article/10.3390/ijerph20021305/s1.

## References

[1] Flegal K.M., Kit B.K., Orpana H., Graubard B.I. (2013). Association of all-cause mortality with overweight and obesity using standard body mass index categories: A systematic review and meta-analysis. JAMA.

[2] Guh D.P., Zhang W., Bansback N., Amarsi Z., Birmingham C.L., Anis A.H. (2009). The incidence of co-morbidities related to obesity and overweight: A systematic review and meta-analysis. BMC Public Health.

[3] Broussard J.L., Van Cauter E. (2016). Disturbances of sleep and circadian rhythms: Novel risk factors for obesity. Curr. Opin. Endocrinol. Diabetes Obes..

[4] Qian J., Scheer F.A. (2016). Circadian system and glucose metabolism: Implications for physiology and disease. Trends Endocrinol. Metab..

[5] Johnston J.D., Ordovás J.M., Scheer F.A., Turek F.W. (2016). Circadian rhythms, metabolism, and chrononutrition in rodents and humans. Adv. Nutr..

[6] Bray M.S., Young M.E. (2007). Circadian rhythms in the development of obesity: Potential role for the circadian clock within the adipocyte. Obes. Rev..

[7] Manenschijn L., van Kruysbergen R.G., de Jong F.H., Koper J.W., van Rossum E.F. (2011). Shift work at young age is associated with elevated long-term cortisol levels and body mass index. J. Clin. Endocrinol. Metab..

[8] Maury E., Ramsey K.M., Bass J. (2010). Circadian rhythms and metabolic syndrome: From experimental genetics to human disease. Circ. Res..

[9] Scheer F.A., Hilton M.F., Mantzoros C.S., Shea S.A. (2009). Adverse metabolic and cardiovascular consequences of circadian misalignment. Proc. Natl. Acad. Sci. USA.

[10] Horne J.A., Östberg O. (1976). A self-assessment questionnaire to determine morningness-eveningness in human circadian rhythms. Int. J. Chronobiol..

[11] Baehr E.K., Revelle W., Eastman C.I. (2000). Individual differences in the phase and amplitude of the human circadian temperature rhythm: With an emphasis on morningness–eveningness. J. Sleep Res..

[12] Bailey S.L., Heitkemper M.M. (2001). Circadian rhythmicity of cortisol and body temperature: Morningness-eveningness effects. Chronobiol. Int..

[13] Urbán R., Magyaródi T., Rigó A. (2011). Morningness-eveningness, chronotypes and health-impairing behaviors in adolescents. Chronobiol. Int..

[14] Arora T., Taheri S. (2015). Associations among late chronotype, body mass index and dietary behaviors in young adolescents. Int. J. Obes..

[15] Sun X., Gustat J., Bertisch S.M., Redline S., Bazzano L. (2020). The association between sleep chronotype and obesity among black and white participants of the Bogalusa Heart Study. Chronobiol. Int..

[16] Knutson K.L., Spiegel K., Penev P., Van Cauter E. (2007). The metabolic consequences of sleep deprivation. Sleep Med. Rev..

[17] Knutson K.L., Van Cauter E. (2008). Associations between sleep loss and increased risk of obesity and diabetes. Ann. N. Y. Acad. Sci..

[18] Krejcie R.V., Morgan D.W. (1970). Determining sample size for research activities. Educ. Psychol. Meas..

[19] World Health Organization (2011). Waist Circumference and Waist-Hip Ratio: Report of a WHO Expert Consultation, Geneva, 8–11 December 2008.

[20] Bouchard C., Tremblay A., Leblanc C., Lortie G., Savard R., Theriault G. (1983). A method to assess energy expenditure in children and adults. Am. J. Clin. Nutr..

[21] Buysse D.J., Reynolds I.I.I.C.F., Monk T.H., Berman S.R., Kupfer D.J. (1989). The Pittsburgh Sleep Quality Index: A new instrument for psychiatric practice and research. Psychiatry Res..

[22] Johns M.W. (1991). A new method for measuring daytime sleepiness: The Epworth sleepiness scale. Sleep.

[23] BaHammam A.S., Almistehi W., Almestehi W., Albatli A., AlShaya S. (2011). Distribution of chronotypes in a large sample of young adult Saudis. Ann. Saudi Med..

[24] Lehnkering H., Siegmund R. (2007). Influence of chronotype, season, and sex of subject on sleep behavior of young adults. Chronobiol. Int..

[25] Fabbri M., Antonietti A., Giorgetti M., Tonetti L., Natale V. (2007). Circadian typology and style of thinking differences. Learn. Individ. Differ..

[26] Lucassen E.A., Zhao X., Rother K.I., Mattingly M.S., Courville A.B., de Jonge L., Csako G., Cizza G., Sleep Extension Study Group (2013). Evening chronotype is associated with changes in eating behavior, more sleep apnea, and increased stress hormones in short sleeping obese individuals. PloS ONE.

[27] Yu J.H., Yun C.-H., Ahn J.H., Suh S., Cho H.J., Lee S.K., Yoo H.J., Seo J.A., Kim S.G., Choi K.M. (2015). Evening chronotype is associated with metabolic disorders and body composition in middle-aged adults. J. Clin. Endocrinol. Metab..

[28] Culnan E., Kloss J.D., Grandner M. (2013). A prospective study of weight gain associated with chronotype among college freshmen. Chronobiol. Int..

[29] Mota M.C., Waterhouse J., De-Souza D.A., Rossato L.T., Silva C.M., Araújo M.B.J., Tufik S., de Mello M.T., Crispim C.A. (2016). Association between chronotype, food intake and physical activity in medical residents. Chronobiol. Int..

[30] Almoosawi S., Vingeliene S., Gachon F., Voortman T., Palla L., Johnston J., Van Dam R.M., Darimont C., Karagounis L.G. (2019). Chronotype: Implications for epidemiologic studies on chrono-nutrition and cardiometabolic health. Adv. Nutr..

[31] Archer E., Lavie C.J., Hill J.O. (2018). The failure to measure dietary intake engendered a fictional discourse on diet-disease relations. Front. Nutr..

[32] Roenneberg T., Allebrandt K.V., Merrow M., Vetter C. (2012). Social jetlag and obesity. Curr. Biol..

[33] Vitale J.A., Roveda E., Montaruli A., Galasso L., Weydahl A., Caumo A., Carandente F. (2015). Chronotype influences activity circadian rhythm and sleep: Differences in sleep quality between weekdays and weekend. Chronobiol. Int..

[34] Bodur M., Bidar N., Yardimci H. (2021). Effect of chronotype on diet and sleep quality in healthy female students: Night lark versus early bird. Nutr. Food Sci..

[35] Rique G.L.N., Fernandes Filho G.M.C., Ferreira A.D.C., de Sousa-Munoz R.L. (2014). Relationship between chronotype and quality of sleep in medical students at the Federal University of Paraiba, Brazil. Sleep Sci..

[36] Upadhyay D., Agrawal S., Verma A., Mahajan N., Shah N. (2019). Evaluation of prevalence of daytime sleepiness and its association with chronotype in undergraduate medical and paramedical students. Natl. J. Integr. Res. Med..

[37] Bayon V., Leger D., Gomez-Merino D., Vecchierini M., Chennaoui M. (2014). Sleep debt and obesity. Ann. Med..

[38] Taheri S. (2006). The link between short sleep duration and obesity: We should recommend more sleep to prevent obesity. Arch. Dis. Child..

[39] Martin J.S., Hébert M., Ledoux É., Gaudreault M., Laberge L. (2012). Relationship of chronotype to sleep, light exposure, and work-related fatigue in student workers. Chronobiol. Int..

[40] Maukonen M., Kanerva N., Partonen T., Kronholm E., Konttinen H., Wennman H., Männistö S. (2016). The associations between chronotype, a healthy diet and obesity. Chronobiol. Int..

[41] Vera B., Dashti H.S., Gómez-Abellán P., Hernández-Martínez A.M., Esteban A., Scheer F.A.J.L., Saxena R., Garaulet M. (2018). Modifiable lifestyle behaviors, but not a genetic risk score, associate with metabolic syndrome in evening chronotypes. Sci. Rep..

[42] Munoz J., Cañavate R., Hernandez C.M., Cara-Salmerón V., Morante J. (2017). The association among chronotype, timing of food intake and food preferences depends on body mass status. Eur. J. Clin. Nutr..

[43] Sato-Mito N., Shibata S., Sasaki S., Sato K. (2011). Dietary intake is associated with human chronotype as assessed by both morningness–eveningness score and preferred midpoint of sleep in young Japanese women. Int. J. Food Sci. Nutr..

[44] Toktas N., Erman K.A., Mert Z. (2018). Nutritional Habits According to Human Chronotype and Nutritional Status of Morningness and Eveningness. J. Educ. Train. Stud..

[45] Maukonen M., Kanerva N., Partonen T., Kronholm E., Tapanainen H., Kontto J., Männistö S. (2017). Chronotype differences in timing of energy and macronutrient intakes: A population-based study in adults. Obesity.

[46] Waterhouse J., Buckley P., Edwards B., Reilly T. (2003). Measurement of, and some reasons for, differences in eating habits between night and day workers. Chronobiol. Int..

