# Supplementary material for: Chronotypes and their Association with Obesity-Related Lifestyle Behaviors among Young Female Adults
Source: Int J Environ Res Public Health. 2023 Jan 11;20(2):1305. doi: 10.3390/ijerph20021305 (PMC9859457; doi:10.3390/ijerph20021305)
Supplement: Supplementary file 1 [file ijerph-20-01305-s001.zip › ijerph-2053392-supplementary.pdf]

Supplementary Materials

Morningness–Eveningness Questionnaire

For each question, please select the answer that best describes you by circling the point value that best indicates how you have felt in recent weeks.

1. *Approximately* what time would you get up if you were entirely free to plan your day?

[5] 5:00 AM–6:30 AM *(05:00–06:30 h)*
[4] 6:30 AM–7:45 AM *(06:30–07:45 h)*
[3] 7:45 AM–9:45 AM *(07:45–09:45 h)*
[2] 9:45 AM–11:00 AM *(09:45–11:00 h)*
[1] 11:00 AM–12 noon *(11:00–12:00 h)*

2. *Approximately* what time would you go to bed if you were entirely free to plan your evening?

[5] 8:00 PM–9:00 PM *(20:00–21:00 h)*
[4] 9:00 PM–10:15 PM *(21:00–22:15 h)*
[3] 10:15 PM–12:30 AM *(22:15–00:30 h)*
[2] 12:30 AM–1:45 AM *(00:30–01:45 h)*
[1] 1:45 AM–3:00 AM *(01:45–03:00 h)*

3. If you usually have to get up at a specific time in the morning, how much do you depend on an alarm clock?

[4] Not at all
[3] Slightly
[2] Somewhat
[1] Very much

4. How easy do you find it to get up in the morning (when you are not awakened unexpectedly)?

[1] Very difficult
[2] Somewhat difficult
[3] Fairly easy
[4] Very easy

5. How alert do you feel during the first half hour after you wake up in the morning?

[1] Not at all alert
[2] Slightly alert
[3] Fairly alert
[4] Very alert

6. How hungry do you feel during the first half hour after you wake up?

[1] Not at all hungry
[2] Slightly hungry
[3] Fairly hungry
[4] Very hungry

7. During the first half hour after you wake up in the morning, how do you feel?

[1] Very tired
[2] Fairly tired
[3] Fairly refreshed
[4] Very refreshed

8. If you had no commitments the next day, what time would you go to bed compared to your usual bedtime?

[4] Seldom or never later
[3] Less that 1 h later
[2] 1–2 h later

[1] More than 2 h later

9. You have decided to do physical exercise. A friend suggests that you do this for one hour twice a week, and the best time for him is between 7 and 8 AM *(07–08 h)*. Bearing in mind nothing but your own internal "clock," how do you think you would perform?

[4] Would be in good form
[3] Would be in reasonable form
[2] Would find it difficult
[1] Would find it very difficult

10. At *approximately* what time in the evening do you feel tired, and as a result in need of sleep?

[5] 8:00 PM–9:00 PM (20:00–21:00 h)
[4] 9:00 PM–10:15 PM (21:00–22:15 h)
[3] 10:15 PM–12:45 AM (22:15–00:45 h)
[2] 12:45 AM–2:00 AM (00:45–02:00 h)
[1] 2:00 AM–3:00 AM (02:00–03:00 h)

11. You want to be at your peak performance for a test that you know is going to be mentally exhausting and will last two hours. You are entirely free to plan your day. Considering only your "internal clock," which one of the four testing times would you choose?

[6] 8 AM–10 AM (08–10 h)
[4] 11 AM–1 PM (11–13 h)
[2] 3 PM–5 PM (15–17 h)
[0] 7 PM–9 PM (19–21 h)

12. If you got into bed at 11 PM *(23 h)*, how tired would you be?

[0] Not at all tired
[2] A little tired
[3] Fairly tired
[5] V ery tired

13. For some reason you have gone to bed several hours later than usual, but there is no need to get up at any particular time the next morning. Which one of the following are you most likely to do?

[4] Will wake up at usual time, but will not fall back asleep
[3] Will wake up at usual time and will doze thereafter
[2] Will wake up at usual time, but will fall asleep again
[1] Will not wake up until later than usual

14. One night you have to remain awake between 4 and 6 AM *(04–06 h)* in order to carry out a night watch. You have no time commitments the next day. Which one of the alternatives would suit you best?

[1] Would not go to bed until the watch is over
[2] Would take a nap before and sleep after
[3] Would take a good sleep before and nap after
[4] Would sleep only before the watch

15. You have two hours of hard physical work. You are entirely free to plan your day. Considering only your internal "clock," which of the following times would you choose?

[4] 8 AM–10 AM (08–10 h)
[3] 11 AM–1 PM (11–13 h)
[2] 3 PM–5 PM (15–17 h)
[1] 7 PM–9 PM (19–21 h)

16. You have decided to do physical exercise. A friend suggests that you do this for one hour twice a week. The best time for her is between 10 and 11 PM *(22–23 h)*. Bearing in mind only your internal "clock," how well do you think you would perform?

[1] Would be in good form
[2] Would be in reasonable form
[3] Would find it difficult
[4] Would find it very difficult

17. Suppose you can choose your own work hours. Assume that you work a five-hour day (including breaks), your job is interesting, and you are paid based on your performance. At *approximately* what time would you choose to begin?

[5] 5 h starting between 4 and 8 AM (04–08 h)
[4] 5 h starting between 8 and 9 AM (08–09 h)
[3] 5 h starting between 9 AM and 2 PM (09–14 h)
[2] 5 h starting between 2 and 5 PM (14–17 h)
[1] 5 h starting between 5 PM and 4 AM (17–04 h)

18. At approximately what time of day do you usually feel your best?

[5] 5–8 AM (05–08 h)
[4] 8–10 AM (08–10 h)
[3] 10 AM–5 PM (10–17 h)
[2] 5–10 PM (17–22 h)
[1] 10 PM–5 AM (22–05 h)

19. One hears about "morning types" and "evening types." Which one of these types do you consider yourself to be?

[6] Definitely a morning type
[4] Rather more a morning type than an evening type
[2] Rather more an evening type than a morning type
[1] Definitely an evening type
